# The Imbibition of Pea (*Pisum sativum* L.) Seeds in Silver Nitrate Reduces Seed Germination, Seedlings Development and Their Metabolic Profile

**DOI:** 10.3390/plants11141877

**Published:** 2022-07-19

**Authors:** Joanna Szablińska-Piernik, Lesław Bernard Lahuta, Karolina Stałanowska, Marcin Horbowicz

**Affiliations:** Department of Plant Physiology, Genetics and Biotechnology, University of Warmia and Mazury in Olsztyn, Oczapowskiego Street 1A, 10-719 Olsztyn, Poland; karolina.stalanowska@uwm.edu.pl (K.S.); marcin.horbowicz@uwm.edu.pl (M.H.)

**Keywords:** pea, seeds, germination, seedlings, silver nanoparticles, silver ions, metabolome

## Abstract

The use of silver nanoparticles (Ag NPs) on plants is accompanied by the occurrence of Ag^+^ ions, so the research of the effects of both on plants should be related. Therefore, in our study, the effects of Ag NPs suspension (containing Ag^0^ at 20 mg/L) and AgNO_3_ solutions (with the concentration of Ag^+^ ions at 20 and 50 mg/L) on the seed germination and early seedling growth (4 days) of pea (*Pisum sativum* L.) were compared. Both Ag NPs and AgNO_3_ did not decrease seed germination, and even stimulated seedling growth. In seedlings developing in the Ag NPs suspension, an increase in monosaccharides, homoserine and malate was noted. In the next experiment, the effect of short-term seed imbibition (8 h) in AgNO_3_ at elevated concentrations, ranging from 100 to 1000 mg/L, on the further seed germination, seedling growth (in absence of AgNO_3_) and their polar metabolic profiles were evaluated. The seed imbibition in AgNO_3_ solutions at 500 and 1000 mg/L reduced seed germination, inhibited seedlings’ growth and caused morphological deformations (twisting and folding of root). The above phytotoxic effects were accompanied by changes in amino acids and soluble carbohydrates profiles, in both sprouts and cotyledons. In deformed sprouts, the content of homoserine and asparagine (major amino acids) decreased, while alanine, glutamic acid, glutamine, proline, GABA (γ-aminobutyric acid) and sucrose increased. The increase in sucrose coincided with a decrease in glucose and fructose. Sprouts, but not cotyledons, also accumulated malic acid and phosphoric acid. Additionally, cotyledons developed from seeds imbibed with AgNO_3_ contained raffinose and stachyose, which were not detectable in sprouts and cotyledons of control seedlings. The obtained results suggest the possible disturbances in the mobilization of primary (oligosaccharides) and presumably major storage materials (starch, proteins) as well as in the primary metabolism of developing seedlings.

## 1. Introduction

The growing interest and use of silver nanoparticles (Ag NPs, particles size ≤ 100 nm) due to their beneficial properties in various industry sectors, e.g., medicine, textile, cosmetics, home appliance, environment, construction, food, printing, agriculture, sports and fitness, electronics and others, also causes their increasing release to the environment [1,2]. Nanoparticles penetrate the environment [1,3] through sewage sludge [4,5], production waste [6], as well as agrochemicals [7,8]. However, Ag NPs in the environment undergo transformations leading to the release of Ag^+^ ions used to produce them. Thus, evaluation of the effects of Ag^+^ ions should accompany or precede studies of the effects of Ag NPs on plants.

In recent years, there has been an increasing number of reports indicating the effects of Ag NPs on various plant species [9,10], including model plants [11,12] and economically important crops [13,14,15]. The effect of Ag NPs on plants depends on their size, shape, coating agents, concentration [16] and released Ag^+^ ions [17,18]. Ag NPs and Ag^+^ ions are taken up from the soil by the roots’ epidermal cells, then are translocated between cells through the plasmodesmata (or apoplastically) and later on via vascular bundles to the shoot [19,20]. Both Ag NPs and Ag^+^ ions can be accumulated in cells, causing oxidative stress [21,22] and disturbances in metabolism [15,23], water homeostasis, chlorophyll biosynthesis [24], mitotic cell division and chromosomal structure [10,25,26]. In some plant species, the application of Ag NPs stimulates their growth, which was found in *Phaseolus vulgaris* and *Zea mays* [27], *Pisum sativum* [28] and *Trigonella foenum-graecum* [29]. The effects of Ag NPs on seeds’ germination are dose- and size-dependent, as was reported in *Oryza sativa* [30], *Triticum aestivum* [15], *Solanum lycopersicum*, *Raphanus sativus* and *Brassica oleracea* [31].

AgNO_3_ has been used in in vitro culture of plants as a plant growth modulator in organogenesis, morphogenesis and other physiological processes [32,33,34,35]. Silver nitrate affects callus induction as well as shoot and root formation in wheat [36]. Licorice (*Glycyrrhiza glabra* L.) seeds after germination in Murashige and Skooge (MS) medium containing AgNO_3_ had increased root, hypocotyl and shoot growth, and decreased stomata and trichomes in 20-day-old seedlings [37]. AgNO_3_ also increased cell divisions in root and shoot tips, decreasing the number of vascular tissues, the epidermal thickness, and intercellular spaces in the mesophyll [37]. Ag^+^ ions applied at low doses also enhanced the growth of cowpea seedlings [38].

Pea (*P. sativum* L.) is one of the most important commercial legume crops used as food for humans and livestock, mainly due to the high protein content in seeds [39]. It is also a model object of legumes for genetic studies [40]. There are only a few reports describing the effect of Ag NPs on germination [26,41], root development [26], as well as on ultrastructural and molecular changes in the leaves and roots of pea seedlings [42]. These studies showed that Ag NPs at low concentration (20 mg/L) did not affect pea germination, whereas at higher levels (80–160 mg/L) they inhibited both germination and seedling growth and caused distinct root deformations. Their genotoxic effects resulted in decreased mitotic index and chromosomal abnormalities [6,26]. Detailed studies have shown that Ag NPs can accumulate in the intercellular spaces between cells as well as in cell walls and plasmodesmata. It was shown that in the roots of pea seedlings, an increasing number of mitochondria deformations occur in response to an increasing concentration of Ag NPs. Moreover, a decreasing number of chloroplasts with deformed shapes was observed in cotyledons of Ag NPs-treated seedlings. A molecular study showed an increase in the activity of some enzymes (α, β esterase and peroxidase) and genotoxic effects of Ag NPs on the genome manifested by DNA damage [42]. Thus, it can be expected that Ag^+^ ions originating from Ag NPs can also affect cell metabolism [3], but evidence for this in pea plants is lacking.

Therefore, in the present study, the effects of Ag NPs and AgNO_3_ on germination and development of pea seedlings and their polar metabolic profiles were initially compared. Both compounds were applied during 4 days of seed germination. Then, the short-term seeds imbibition in AgNO_3_ at high concentrations (100–500 and 1000 mg/L) on the further seed germination and seedlings’ growth (in absence of AgNO_3_) of ten pea cultivars were evaluated to find the toxic concentrations of AgNO_3_. Finally, the study focused on the effect of Ag+ ions on the primary metabolism in pea seedlings of the Nemo cultivar.

## 2. Results

### 2.1. The Effect of Ag NPs and AgNO_3_ on Germination, Seedling Development and Metabolic Profile of Pea

**Figure 1:**
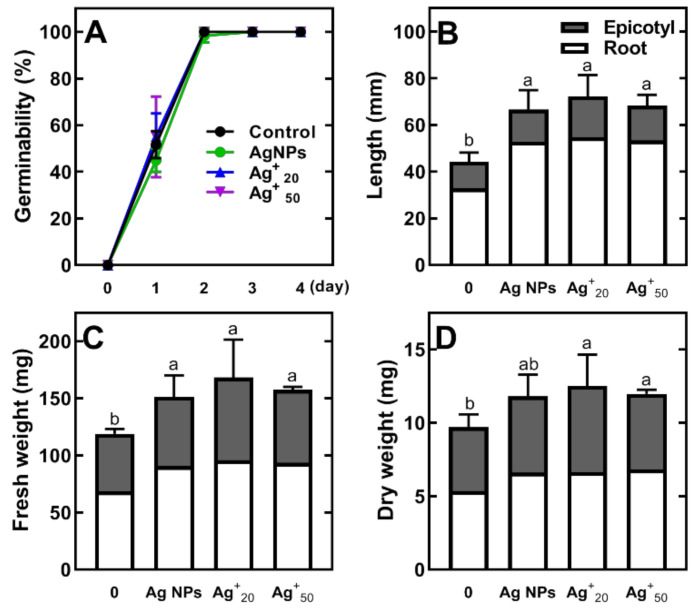
The effect of seed imbibition with Ag NPs at 20 mg/L and Ag^+^ ions at concentrations of 20 and 50 mg/L on seed germinability (**A**), length (**B**), fresh weight (**C**) and dry weight (**D**) of 4-day-old pea (cv. Tarchalska) seedlings. Results are means (*n* = 3) + SD. The same letters (a,b) above the bars (on **B**–**D**) indicate no significant (*p* < 0.05) differences according to ANOVA and Tukey post-hoc test for seedlings’ (root and epicotyl) length, fresh and dry weight.

Silver nanoparticles (Ag NPs) at a concentration of 20 mg/L and AgNO_3_ at 20 and 50 mg/L concentrations of Ag^+^ ions did not affect germination of pea seeds (Figure 1A). However, these solutions significantly stimulated elongation of seedlings’ length (root and epicotyl, Figure 1B), as well as their fresh weight (FW) and dry weight (DW) (Figure 1C,D).

In seedlings, 42 polar metabolites were identified (Table S1). There were soluble carbohydrates (9), amino acids (20), organic acids (11) and other compounds (2). The major polar metabolites were soluble carbohydrates and amino acids, sharing together ca. 80% of total identified polar metabolites (TIPMs). In cotyledons, most acids (acetic, butyric, oxoglutaric, oxalic, aspartic, erythronic and gluconic) were below detection limits. Besides, cotyledons contained traces of raffinose and stachyose, which were not detected in roots and epicotyls. In control seedlings, sucrose was the quantitatively major sugar (25.6, 37.4 and 47.0 mg/g DW, in root, epicotyl and cotyledons, respectively), whereas homoserine dominated among amino acids (28.1, 26.9 and 2.1 mg/g DW) and asparagine among protein amino acids (3.9 and 4.2 mg/g DW, in root and epicotyl, respectively). The tricarboxylic acid cycle (TCA) intermediates (citrate and malate) were a major fraction of organic acids. Tissues of roots and epicotyls also contained a considerable amount of phosphoric acid (ca. 8 mg/g DW).

**Figure 2:**
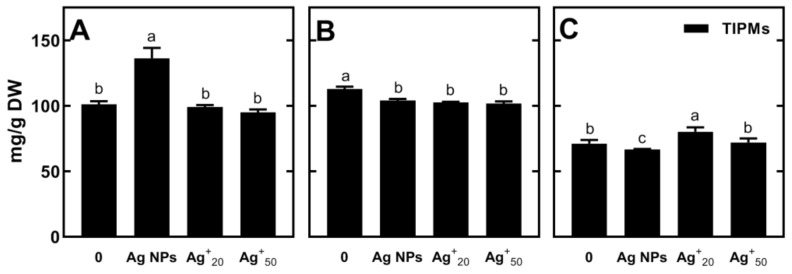
The total contents of identified polar metabolites (TIPMs) in the roots (**A**), epicotyls (**B**) and cotyledons (**C**) of 4-day-old pea seedlings (cv. Tarchalska) developing in double-distilled water (0), Ag NPs suspension (20 mg/L) and AgNO_3_ solutions (20 and 50 mg/L of Ag^+^ ions). Results are means (*n* = 3) + SD. The same letters (a–c) above the bars indicate no significant (*p* < 0.05) differences according to ANOVA and Tukey post-hoc test.

Despite this seedling growth stimulation by both Ag NPs and AgNO_3_, only slight changes occurred in polar metabolites. Regardless of the seed’s treatment, the concentration of TIPMs was higher in the root and epicotyl than that in the cotyledons (Figure 2, Tables S2–S4). In the roots developing from seeds in the presence of Ag NPs, the content of TIPMs was significantly higher than that in the roots of seedlings developed in AgNO_3_ (Figure 2A, Table S2).

In epicotyls, both Ag NPs and AgNO_3_ caused a decrease in TIPMs (Figure 2B, Table S3). In cotyledons, however, Ag NPs decreased TIPMs, while the AgNO_3_ solution at the concentration of Ag^+^ ions equal to that of Ag NPs (20 mg/L) led to an increase in TIPMs (Figure 2C, Table S4). Under Ag NPs-treatment, the content of total monosaccharides (glucose, fructose and galactose) in roots increased four-fold (up to 23.73 mg/g DW), without any changes in the level of sucrose, a predominant sugar (Table S2). In effect, the content of total soluble carbohydrates (TSCs) increased from 33.51 to 50.97 mg/g DW. The treatment with AgNO_3_ and Ag NPs also increased the content of homoserine and malate in roots (an increase from 28.11 to 45.12 mg/g DW and from 5.84 to 10.25 mg/g DW, respectively). On the other hand, in epicotyl, the decrease in TIPMs was due to a drastic reduction in sucrose (from ca. 38 mg/g DW in control, to 20–25 mg/g DW in Ag NPs and AgNO_3_-treated seedlings, Table S3).

### 2.2. The Effect of Short-Term Seed Imbibition in AgNO_3_ Solutions on Germination and Seedling Development of Pea

**Figure 3:**
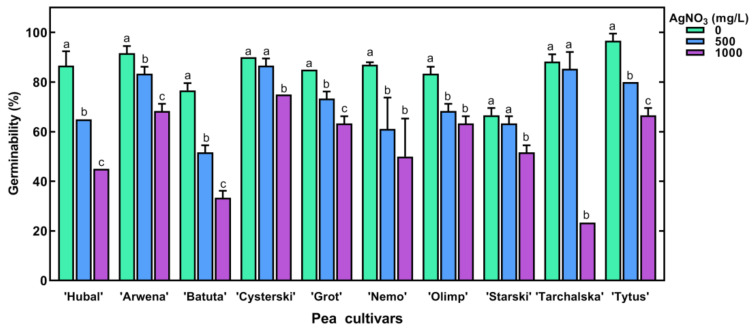
The effect of seed imbibition of ten pea cultivars in AgNO_3_ at concentrations of 0, 500 and 1000 mg/L for 8 h on their germinability. Results are means (*n* = 3) + SD. The same letters (a–c) above the bars indicate no significant (*p* < 0.05) differences according to ANOVA and Tukey post-hoc test.

The imbibition of pea seeds in AgNO_3_ at concentrations of 100 and 250 mg/L for 8 h did not affect seeds’ germinability (ca. 90–100%) and seedling development (Table S5). However, AgNO_3_ at 1000 mg/L decreased the germination of all tested pea cultivars (Figure 3).

The effect of AgNO_3_ on seedling growth was cultivar-dependent (Figure S1). Although the length of seedling significantly decreased in six cultivars (‘Batuta’, ‘Cysterski’, ‘Grot’, ‘Nemo’, ‘Olimp’, and ‘Tarchalska’), only in ‘Nemo’ the was decrease in seedling length, according to increasing concentrations of AgNO_3_, accompanied with a decrease in DW (Figure S1). Moreover, ca. 35–40% of ‘Nemo’ seedlings were short and dramatically deformed: epicotyl remained hooked, while the root was twisted, folded, and hooked (Figure S2). Therefore, we compared the metabolic profiles of those sprouts with the profiles of normal developing seedlings (in root and epicotyl, together).

### 2.3. Changes in Metabolic Profiles of Pea Seedlings

#### 2.3.1. Principal Component Analysis

**Figure 4:**
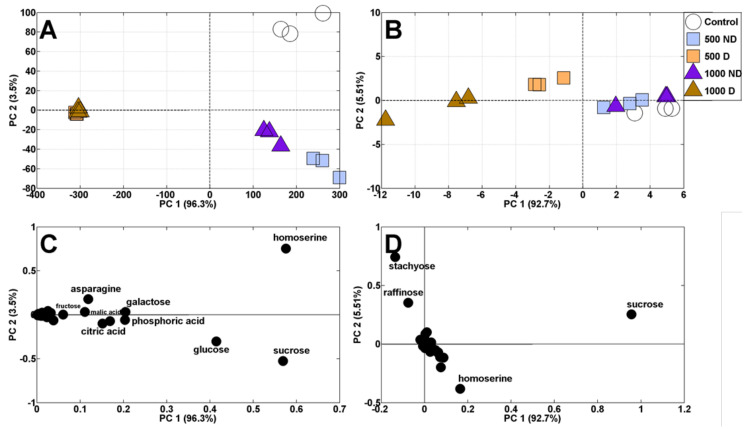
PCA profiles of polar metabolites in sprouts (**A**) and cotyledons (**B**) of 4-day-old pea seedlings from seeds imbibed in water (Control) or AgNO_3_ at concentrations of 500 and 1000 mg/L. The non-deformed and deformed sprouts were signed as ND and D, respectively. The PCA loading plots of the polar metabolites in sprouts and cotyledons are present on (**C**,**D**), respectively.

In control seedlings of pea cv. Nemo, the same polar metabolites were identified as in previously analyzed seedlings of cv. Tarchalska (Table S1). Principal component analysis (PCA) of polar metabolites revealed a shift in the distribution of samples of both sprouts and cotyledons (Figure 4A,B). According to high PC1 scores (96.3 and 92.7% of variability for sprouts and cotyledons, respectively), samples of deformed seedlings were clearly separated from samples of those with normal morphology (Figure 4A,B). Additionally, control sprouts were separated from those developing from seeds imbibed in AgNO_3_ solutions (PC2, sharing 3.5% of variability, Figure 4A). The distribution of sprouts samples resulted mainly from changes in the contents of homoserine, sucrose and glucose (Figure 4C), whereas the distribution of cotyledons samples was related to sucrose, homoserine, raffinose and stachyose (Figure 4D).

#### 2.3.2. Changes in the Concentration of Polar Metabolites

The content of TIPMs was much higher in sprouts than in cotyledons, regardless of the concentration of AgNO_3_ solutions used to imbibe the seeds. The content of TSCs was as high as in sprouts and cotyledons (51.24 and 48.61 mg/g DW, respectively). However, the content of total amino acids (TAAs) and total organic acids (TOAs) in sprouts was ca. three- and five-fold higher than that in cotyledons, respectively (Tables S6 and S7).

**Figure 5:**
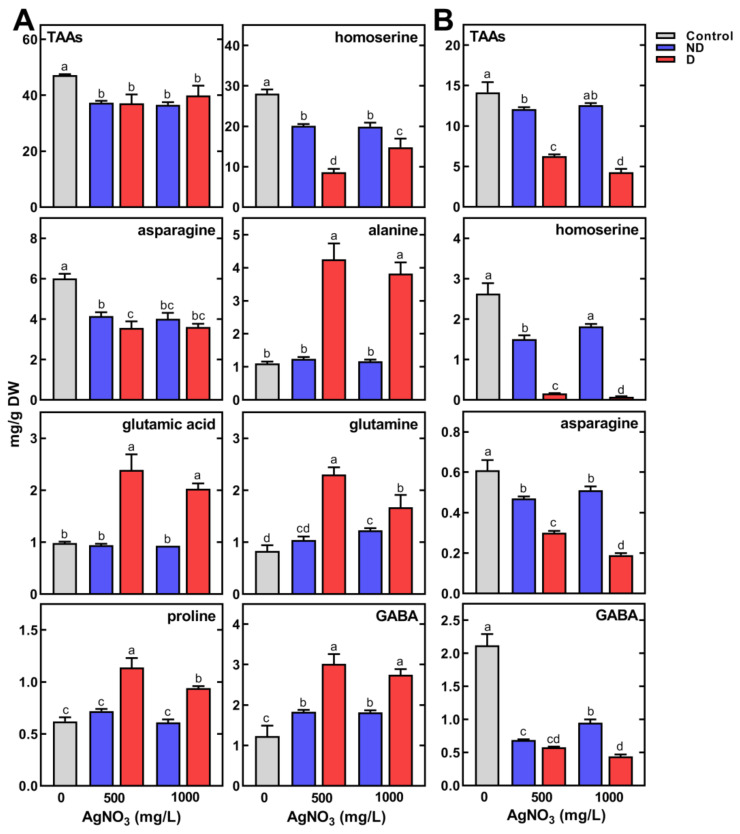
The effect of pea seeds’ imbibition for 8 h with AgNO_3_ at concentrations of 0, 500 and 1000 mg/L on the content of total amino acids (TAAs) and selected free amino acids in control, non-deformed (ND) and deformed (D) sprouts (**A**) and cotyledons (**B**) of 4-day-old seedlings. Results are means (*n* = 3) + SD. Bars marked with the same letters (a–d) are not significantly different after one-way ANOVA and Tukey post hoc test.

The inhibition of seedling development following seed imbibition by AgNO_3_ at 500 mg/L and 1000 mg/L was accompanied by a significant reduction in TAAs in both sprouts and cotyledons (Figure 5A,B). Moreover, the cotyledons of deformed seedlings contained ca. two- to three-fold less TAAs than the cotyledons of non-deformed seedlings (Figure 5B).

The contents of major amino acids in the control sprouts, being homoserine and asparagine, were 28.11 and 6.01 mg/g DW, respectively. Their content in non-deformed sprouts, developing from seeds imbibed in a high concentration of AgNO_3_ (500 and 1000 mg/L), decreased to 20.13 and 19.92 and to 4.15 and 4.01 mg/g DW. In contrast, these contents decreased to 8.66 and 14.83 and to 3.56 and 3.60 mg/g DW in deformed sprouts, respectively (Figure 5A). The opposite response was found in the accumulation of alanine, glutamic acid, glutamine, proline and γ-aminobutyric acid (GABA, Figure 5A). Similar changes to those shown in Figure 5A were found in the contents of less abundant amino acids, i.e., arginine, hydroxyproline, isoleucine and lysine (Table S6). It is interesting to note that the content of these amino acids was significantly higher in the deformed sprouts than in those with normal morphology. Contrary to sprouts, the contents of TAAs in cotyledons of deformed seedlings were two- to three-fold lower compared to the control (Figure 5B). This was mainly due to the decrease in homoserine, asparagine, GABA (Figure 5B) and, to a lesser extent, lysine and proline (Table S6).

**Figure 6:**
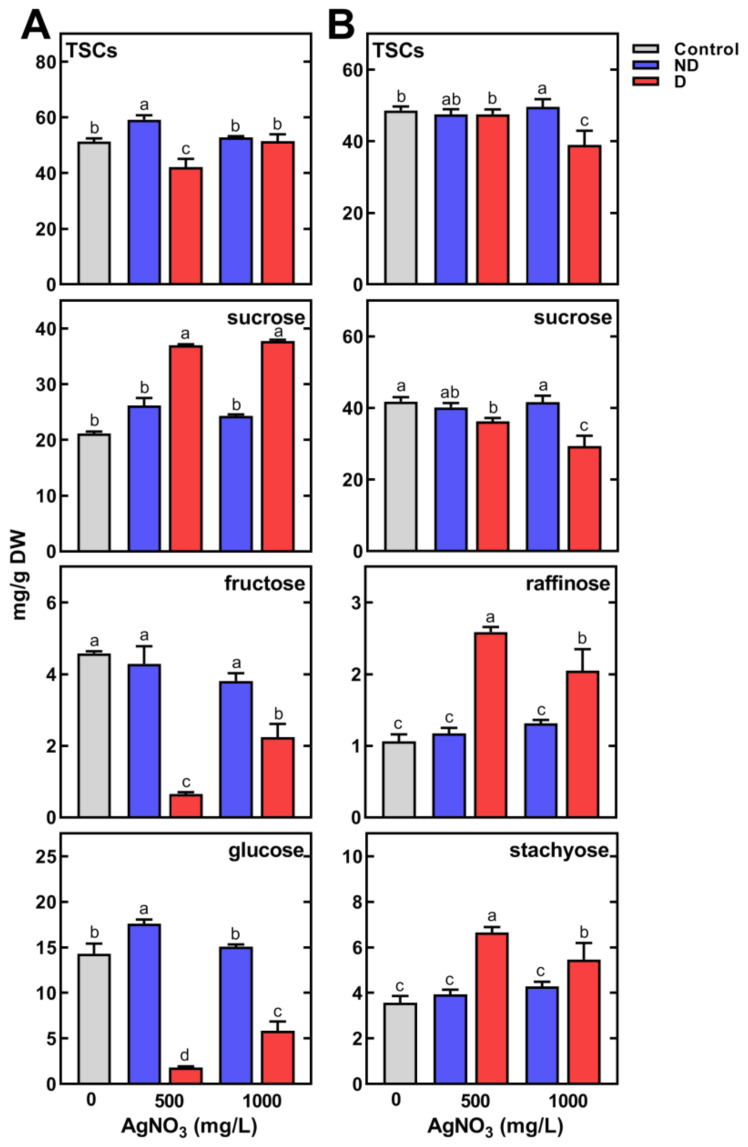
The effect of pea seeds imbibition for 8 h with AgNO_3_ at concentrations of 0, 500 and 1000 mg/L on the content of total soluble carbohydrates (TSCs) and selected carbohydrates in control, non-deformed (ND) and deformed (D) sprouts (**A**) and cotyledons (**B**) of 4-day-old seedlings. Results are means (*n* = 3) + SD. Bars marked with the same letters (a–d) are not significantly different after one-way ANOVA and Tukey post hoc test.

In control seedlings, soluble carbohydrates represented ca. 42 and 72% of TIPMs in sprouts and cotyledons, respectively (Table S6 and S7). Considerable changes in TSCs in sprouts were shown only after seeds’ imbibition in AgNO_3_ at a concentration of 500 mg/L. In non-deformed sprouts, TSCs were higher (59.22 mg/g DW) than in the control (51.24 mg/g DW), while in deformed ones TSCs reached a level of 42.16 mg/g DW (Figure 6A).

The content of major carbohydrates, glucose and sucrose, did not change significantly in AgNO_3_-treated normal sprouts. However, in deformed sprouts sucrose content was higher than in non-deformed and control sprouts, while glucose and fructose contents were substantially lower (Figure 6A). A similar decreasing pattern was noted for galactose (Table S6). In cotyledons, TSCs were lower only in deformed seedlings (Figure 6B), due to a decrease in sucrose. However, the contents of raffinose and stachyose were much higher than in normal sprouts obtained from seeds imbibed in water or AgNO_3_ (Figure 6B).

**Figure 7:**
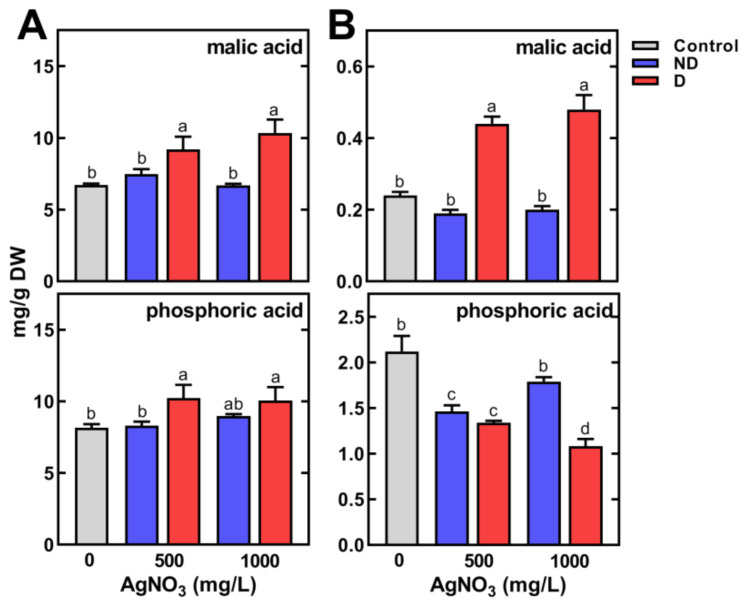
The effect of pea seeds’ imbibition for 8 h with AgNO_3_ at concentrations of 0, 500 and 1000 mg/L on the content of malic and phosphoric acid in control, non-deformed (ND) and deformed (D) sprouts (**A**) and cotyledons (**B**) of 4-day-old seedlings. Results are means (*n* = 3) + SD. Bars marked with the same letters (a–d) are not significantly (*p* < 0.05) different after one-way ANOVA and Tukey post hoc test.

In the case of other polar metabolites, significantly higher contents of malate were noted in sprouts (10.35 mg/g DW) and in cotyledons (0.48 mg/g DW) of deformed seedlings compared to the control (Figure 7).

The phosphate was present at a considerably high level in control seedlings (8.14 and 2.12 mg/g DW in sprouts (Figure 7A) and cotyledons (Figure 7B), respectively). The use of AgNO_3_ increased its content to 10.21 mg/g DW in sprouts and decreased in cotyledons (1.08 mg/g DW).

## 3. Discussion

### 3.1. Pea Response to Ag NPs and Ag^+^ Ions

The application of Ag NPs affects seed germination, which is mainly dependent on the dose and size of nanoparticles [6,43,44]. So far, studies on the effects of Ag NPs on germination of legume seeds have shown mainly negative effects manifested by retardation of pea seed germination [26] or decrease in the germination of lupine seeds [23] and faba bean [45] with increasing Ag NPs concentration. However, the use of a very low dose of Ag NPs (0.25 and 1.25 mg/L) had an immediate beneficial effect, resulting in fast and uniform germination in laboratory and field conditions of bean seeds [46]. In our study, the use of Ag NPs (diameter 10 nm, 20 mg/L), as well as AgNO_3_ with an equal or much higher concentration of Ag^+^ ions (20 and 50 mg/L), did not negatively affect seed germination. Additionally, they stimulated the early growth of the seedling (Figure 1). However, changes in the metabolic profiles of seedlings were different. Nanoparticles stimulated the accumulation of monosaccharides, homoserine and malate in root and in epicotyl, but to a lesser extent. Such changes were not noted in response to Ag^+^ ions. Nonetheless, the comparable growth of seedlings means that at applied concentrations both Ag NPs and Ag^+^ ions did not negatively affect primary metabolism, and presumably mobilization of reserves. However, the further metabolic changes during prolonged seedling growth remain to be evaluated.

### 3.2. Pea Response to AgNO_3_

Although the short-term imbibition of pea seeds in AgNO_3_ solutions at low concentrations (100 and 250 mg/L) had no effect on seed germination, AgNO_3_ at 500 and 1000 mg/L clearly reduced the process as well as seedlings’ growth. Similar results were also demonstrated in previous studies on the effect of silver nanoparticles on the germination of *Cucurbita pepo* [17], *A. thaliana* [19], *O. sativa* [30] and *Vicia faba* [45]. The differential sensitivity of pea cultivars to AgNO_3_, demonstrated in our study (Figure S1) indicates that differences could be expected also among cultivars of one species. The reasons for this effect need further investigation.

The changes of root morphology in pea seedlings developed from seeds imbibed with AgNO_3_ at 500 and 1000 mg/L (Figure S2) were similar to those observed for Ag NPs by Labeeb et al. [26]. In their study, Ag NPs were applied at low concentrations (80–160 mg/L) but for a much longer time (14 days). In our study, Ag^+^ ions at about four times higher concentration were used (and for a shorter period) than the concentration of Ag NPs in the mentioned work. Thus, the phytotoxicity of Ag NPs seems to be much higher than that of Ag^+^ ions. Similar results were obtained previously in studies on *A. thaliana* [11,24] and *Hordeum vulgare* [47], for which Ag NPs had stronger inhibitory effect than Ag^+^, manifested in greater root damage and its inhibited growth. Additionally, previously it was shown that silver nitrate affects callus induction as well as shoot and root formation in wheat [36]. AgNO_3_ also accelerated the growth of licorice seedlings [37] but at low doses also enhanced the growth of pea seedlings [38]. The results obtained in our study show that high concentrations of AgNO_3_ had toxic effects on growing pea seedlings.

#### 3.2.1. Profile of Polar Metabolites in Control Pea Seedlings

The much higher contents of quantitatively main polar metabolites (sucrose, glucose, homoserine, asparagine, malic and citric acid) in pea sprouts than in cotyledons (Table S6) presumably determines the maintenance of high cell turgor, which enables elongation of root and epicotyl [48]. On the other hand, the high concentration of these metabolites may also indicate their crucial role as the major sources of carbon skeletons and energy sources providing the metabolism of growing cells/tissues. Homoserine is a characteristic amino acid for pea seedlings, which content is very low in dry seeds, while rapidly increasing during seedlings’ growth [49] and becomes the main non-protein amino acid in vegetative tissues of pea plants [15,50]. The high contents of homoserine, asparagine and sucrose confirm their role as the main transport forms of nitrogen and carbon in pea seedlings [51,52].

In dry pea seeds, the major sugars are sucrose and its galactosides (raffinose, stachyose and verbascose, called the raffinose family of oligosaccharides, RFOs) [53]. Their content in the embryonic axis is higher than in the cotyledons and decreases rapidly during seed germination [54]. In the axes of pea and soybean, RFOs are completely hydrolyzed within the first 48 h from the start of the seeds’ imbibition, whereas hydrolysis of RFOs in cotyledons extends to 6–7 days [55]. The absence of RFOs in sprouts of 4-day-old seedlings, observed in our study (Table S6), corresponds with the data presented above. Moreover, raffinose and stachyose were still present in the cotyledons, as it was expected. In addition to sucrose and RFOs, the cotyledons also contained significant amounts of *myo*-inositol and traces of monosaccharides (<0.2 mg/g DW). Hydrolysis of RFOs results in the release of galactose and sucrose, which are used to maintain the osmotic potential in the cells of growing sprouts [56] and acts as signaling molecules for sprout elongation [57]. Besides, galactose, when oxidized to galacturonic acid, is the substrate for pectin synthesis [58], which enables cell division and the production of cell wall components during cell elongation [59].

#### 3.2.2. Effect of AgNO_3_ on Metabolic Profiles of Pea Seedlings

The short-term imbibition of pea seeds in AgNO_3_ affected the content of some amino acids in developing seedlings. There were significantly higher decreases in homoserine and asparagine content in deformed seedlings than in those with normal morphology (Figure 5). Homoserine is assumed to be formed from aspartic acid [60,61] and enables nitrogen transport from storage tissues to the growing seedling [51]. A reduction in homoserine content found in our study in both sprouts and cotyledons may indicate inhibition of its biosynthesis. Additionally, the decrease in its content in cotyledons may be related to a deficiency of its precursor, aspartic acid (Table S7). Moreover, the reduction in total free amino acid content in cotyledons may indicate a disruption in their release from degraded storage proteins. In contrast, the increasing content of protein amino acids in sprouts (Figure 5A) may be due to their lack of utilization for the synthesis of some structural proteins. This may be due to both the inhibition of growth and sprout deformation. A previous study has shown in wheat seedlings treated with Ag NPs that expression of several proteins mainly involved in primary metabolism and cell defense are altered [62].

Since silver causes oxidative stress in plant cells [15,63], proline and GABA can be accumulated as protective compounds, maintaining redox balance [64] and functioning as antioxidants [65]. Indeed, our results confirm an accumulation of proline and GABA in sprouts (two-fold higher in deformed seedlings than in normal, Figure 5A). The protective role of proline metabolism is to maintain the intracellular redox potential by regulating the normal level of NADP^+^/NADPH in the cytosol, as well as the ability to bind ^1^O_2_ [66,67]. GABA appears to participate in stress protection by reduction of oxidative damage by stimulating antioxidants [68].

The elevated level of sucrose in deformed sprouts (Figure 6A) was also found earlier in pea seedlings under osmotic stress [69], dehydration [70], as well as in pea plants’ response to soil drought [15,50]. Noticeably, the accumulation of sucrose was accompanied with a decrease in the content of glucose and fructose, presumably used for its synthesis (Figure 6). It is also possible that the decline in the content of monosaccharides was due to their use in respiratory processes in response to abiotic stress [71]. Moreover, this supposition can be confirmed by the higher content of RFOs in the cotyledons of deformed seedlings, as a result of inhibition of RFOs hydrolysis, a process releasing monosaccharides, transported into growing seedlings [54,72].

The content of malic acid, the major organic acid, was higher in both sprouts and cotyledons of deformed seedlings compared to morphologically normal ones. Previous studies have shown accumulation of malic acid in leaves [73] and shoots [15] of pea in response to drought. However, the water content in pea seedlings (both control and developed from AgNO_3_-treated seeds) was constant (90 and 60% in sprouts and cotyledons, respectively), thus the osmotic imbalance appears to be questionable. It is considered that malic acid and sucrose are involved in the osmotic regulation of cells [74], as well as in maintaining redox homeostasis under stress conditions [75]. The malate valve allows for a balance of the ATP/NAD(P)H in subcellular compartments (chloroplasts, mitochondria and peroxisomes), which allows plants to adapt to various stresses [75]. Thus, the similarity in sucrose and malate accumulation under AgNO_3_ treatment, and water/osmotic stresses [69,70], lead us to the suggestion that the same signal/s are presumably triggered under both stresses [76].

On the other hand, organic acids (e.g., citrate, malate) can chelate metal ions, including heavy metal ions, participating in their accumulation/transport in plants [77,78], as well as detoxification [79]. Moreover, some organic acids released from seeds during imbibition/germination can affect ion uptake. Additionally, inside plants, Ag^+^ ions can be reduced to Ag NPs, with reducing monosaccharides, ascorbic acid/citric acid, present in cells as it was revealed by Marchiol et al. [80]. However, it is still unknown if such interactions take place for Ag^+^ ions in pea seedlings.

Another distinct feature found in our study was the high phosphoric acid content in pea sprouts. In the dry seeds, the phosphate is present in a bound form to *myo*-inositol as phytin. During germination, phytin or phytates are a source of *myo*-inositol, phosphate, K, Mg, Ca, Mn, Fe, and Zn which are released by the action of phytase enzymes and then translocated to developing seedlings [81]. In our study, a decrease in the content of phosphate in cotyledons and its increase in sprouts was shown. Moreover, its content increased in sprouts under the influence of AgNO_3_ associated with seedling deformations. So far, the involvement of phosphoric acid in pea’s response to environmental stresses remains unclear. In addition to the presence in the form of phytic acid, phosphorus compounds play a crucial role in plants’ metabolism as sugar phosphates as well as phospholipids and nucleotides [82].

## 4. Materials and Methods

### 4.1. The Effect of Ag NPs and AgNO_3_ on Seed Germination and Seedling Development of Pea

Seeds of pea (*Pisum sativum* L.) cv. Tarchalska (for general purpose, obtained from DANKO Plant Breeding, Poland) were germinated on Petri dishes (ø 10 cm, 20 seeds per dish, in each of 3 replicates) in double-distilled water (control) and a suspension of Ag NPs (Sigma-Aldrich, Saint Louis, MO, USA) with a size of 10 nm (at a concentration of 20 mg/L) or in AgNO_3_ (Sigma-Aldrich, Saint Louis, MO, USA) solutions (at 31.5 mg/L and 78.74 mg/L, corresponding to concentrations of 20 and 50 mg/L of Ag^+^ ions) for 4 days in the darkness (at 22 °C, in climatic chamber ILW 115-T STD, Pol-Eko-Aparatura, Wodzisław Śląski, Poland). After every 24 h of germination, the number of germinated seeds was scored. After 4 days, the length of root and epicotyl and fresh weight of root, epicotyl and cotyledons of seedlings from 3 replicates were measured, and tissues were frozen in liquid nitrogen and stored in an ultra-refrigerator (at −80 °C) for 2 days. Then, samples were freeze-dried (shelf freeze-dryer, Alpha 1–2 LD, Martin Christ, Osterode am Harz, Germany) for 48 h, and dry tissues were pulverized (for 2 min at 22 Hz) in a mixed mill (MM 200, Retsch, Verder Scientific GmbH, Haan, Germany) for extraction of polar metabolites.

### 4.2. The Effect of Short-Term Seeds’ Imbibition with AgNO_3_ on Germination and Seedling Development of Pea

Seeds of ten pea cultivars (one fodder ‘Hubal’ and 9 for general-purpose: ‘Arwena’, ‘Batuta’, ‘Cysterski’, ‘Grot’, ‘Nemo’, ‘Olimp’, ‘Starski’, ‘Tarchalska’, and ‘Tytus’), obtained from DANKO and Poznan Plant Breeding Companies (Poland), were imbibed on Petri dishes (ø 10 cm, 20 seeds per dish) in double-distilled water (control) and AgNO_3_ (Sigma-Aldrich, Saint Louis, MO, USA) solutions (at concentrations of 500 and 1000 mg/L, 20 mL per dish, in each of 3 replicates) for 8 h. Then, seeds from each replicate were placed on double-folded sheets of filter paper (Eurochem BDG, Tarnów, Poland) wetted with double-distilled water, rolled, and transferred into 250 mL glass cylinders. After 4 days of incubation at 22 °C in the darkness, the length of root and epicotyl and fresh weight of root, epicotyl, and cotyledons from 3 replicates were measured and tissues were dried at 80 °C for 24 h to estimate their dry weight.

The control seedlings (sprouts and cotyledons) of cv. Nemo, and those developing from seeds imbibed in AgNO_3_ at concentrations of 500 and 1000 mg/L causing a decrease in sprout (root and epicotyl) growth were used for polar metabolites profiling. Moreover, sprouts developed from seeds imbibed in AgNO_3_ were separated into two groups—sprouts of typical morphology (normal) and those with visible deformations (deformed). Sprouts and cotyledons were collected separately, frozen in liquid nitrogen, stored in an ultra-refrigerator (at −80 °C) for 7 days, and then freeze-dried (shelf freeze-dryer, Alpha 1–2 LD, Martin Christ, Osterode am Harz, Germany) for 48 h.

### 4.3. Polar Metabolite Profiling

Polar metabolites were extracted from freeze-dried and pulverized roots, epicotyls and cotyledons of seedlings cv. Tarchalska (Section 4.1) and sprouts and cotyledons of seedlings cv. Nemo (Section 4.2). The polar metabolites were extracted from 40 mg of tissues (from 3 biological replicates) with 900 μL of the mixture of methanol: water (1:1, *v/v*, containing 100 μg of ribitol as internal standard) at 70 °C for 30 min. Homogenates were centrifuged (20,000× *g* at 4 °C for 20 min) and aliquots of clear supernatant (600 μL) were mixed with 400 μL of cold chloroform to remove non-polar compounds. After centrifugation, the polar fraction was concentrated to dryness in a speed vacuum rotary evaporator (JWElectronic, Warsaw, Poland). The metabolites were derivatized with 40 μL of *O*-methoxamine hydrochloride at a concentration of 20 mg/mL in pyridine by heating at 37 °C for 75 min with continuous shaking (500 rpm). Then, 160 μL of a mixture of MSTFA (*N*-methyl-*N*-trimethylsilyl-trifluoroacetamide) with pyridine (1:1, *v/v*) was added and the mixture was heated at 70 °C for 30 min [83]. The mixtures of TMS (tri-methylsilyl)-derivatives were separated on a ZEBRON ZB-5MSi Guardian capillary column (Phenomenex, Torrance, CA, USA). Metabolites were identified by comparison of their retention time (RT), retention indices (RI, determined according to the saturated hydrocarbons) and mass spectra of original standards derived from Sigma-Aldrich (Sigma-Aldrich, Saint Louis, MO, USA) and from the NIST 05 library (National Institute of Standards and Technology, NIST, Gaithersburg, MD, USA, Table S1). The concentration of metabolites was calculated according to the method described previously [50]. Briefly, two gas chromatographs were used—GC-2030 Nexis (Shimadzu, Kyoto, Japan) equipped with a flame ionization detector (FID) and GC-2010 coupled with a quadrupole mass spectrometry (MS) analyzer (GCMS-QP2010 Plus, Shimadzu, Kyoto, Japan). The same parameters for chromatographic separation were applied in GC-FID and GC-MS analyses. The column oven temperature was programmed as follows: initial temperature 70 °C increased by 20 °C/min to 130 °C, by 8 °C/min to 210 °C, by 3 °C/min to 220 °C, by 10 °C/min to 300 °C (held at this temperature for 3 min) and finally increased again by 20 °C/min to 335 °C (and held for 15.92 min). The temperature of the flame-ionized detector (FID) was 350 °C. The samples were subjected to the split method (10:1) and the injection temperature was 280 °C. In GC-MS, the interface temperature was 280 °C, the ion source temperature 250 °C. The spectra were scanned in a detector from 3.4–45 min, in a range from 50 to 600 *m/z*. Data were collected and analyzed using GC-MS Solution and LabSolutions software (Shimadzu, Kyoto, Japan). For the quantitative calculation of the content of identified polar metabolites, only the data obtained for the peak areas from the GC-FID analyses were used.

### 4.4. Statistical Analysis

The results are means of 3 independent replicates and they were subjected to analysis of variance using a one-way ANOVA and Tukey’s post hoc test (if overall *p* < 0.05) for multiple comparisons in GraphPad Prism and Statistica. Principal component analysis (PCA) for multivariate statistics was performed using COVAIN [84], a MATLAB toolbox including a graphical user interface (MATLAB version 2013a; Math Works, Natick, MA, USA).

## 5. Conclusions

Low concentrations of Ag NPs and AgNO_3_ applied for pea seeds’ imbibition did not affect germination and can stimulate early seedling development. However, high concentrations of AgNO_3_ are toxic to germination and seedling development processes and disrupt primary metabolism, revealed by metabolite profiling. The accumulation of sucrose in the sprout and its galactosides (RFOs) in the cotyledons, as well as glutamate/glutamic acid, alanine, malic acid and phosphoric acid in the sprouts at an early stage of development indicates a strong disturbance in carbon and nitrogen metabolism, and presumably impaired mobilization of storage compounds. Moreover, AgNO_3_ can cause oxidative stress resulting in increased accumulation of proline and GABA in pea sprouts. It seems that the effect of Ag^+^ ions is related to changes in the proteome and transcriptome, which may be the subject of future research. The results also indicate that the phytotoxicity of Ag NPs to pea seeds/seedlings seems not to be determined by the release of Ag^+^ ions from them. However, further study is needed to verify this hypothesis.

## Data Availability

Not applicable.

## Supplementary Materials

The following supporting information can be downloaded at: https://www.mdpi.com/article/10.3390/plants11141877/s1, Figure S1. The effect of seeds imbibition in AgNO3 solutions (at concentrations of 500 mg/L and 1000 mg/L, for 8 h) on the length (A) and dry weight (B) of root (R) and epicotyl (E) of 4-day-old seedling of ten pea cultivars (including one fodder—‘Hubal’ and nine for general purpose). Values are means (*n* = 3) + SD. Bars with the same letters (a–c) are not significantly (*p* < 0.05) different (for a whole seedling) after ANOVA test and Tukey post hoc test. Figure S2. The effect of short-term imbibition of pea (*Pisum sativum* L. cv. Nemo) seeds in AgNO3 solutions (at 500 mg/L and 1000 mg/L for 8 h) on the morphology of 4-day-old seedlings. Control seedlings developed from seeds imbibed in double-distilled water. Normal, non-deformed seedlings—upper panel, deformed—lower. Table S1. The parameters of polar metabolites identification in GC-MS analyses. RT—retention time, RI—retention index, ID—a type of identification of each metabolite—using mass spectra of original standards (S) or mass spectra from NIST 05 library (L), P—a percentage of similarity of identified metabolite to the standard metabolite and/or metabolite from the NIST library. Table S2. The effect of Ag NPs (at 20 mg/L) and AgNO3 solutions (at concentrations corresponding to 20 mg/L and 50 mg/L of Ag+ ions) on the content of polar metabolites in roots of 4-day-old seedlings of pea (*Pisum sativum* L., cv. Tarchalska). Means of 3 replicates. The same letters by the values (a–d) indicate no statistically significant differences (*p* < 0.05) based on ANOVA analysis and Tukey post-hoc test. Table S3. The effect of Ag NPs (at 20 mg/L) and AgNO3 solutions (at concentrations corresponding to 20 mg/L and 50 mg/L of Ag+ ions) on the content of polar metabolites in epicotyls of 4-day-old seedlings of pea (*Pisum sativum* L., cv. Tarchalska). Means of 3 replicates. The same letters by the values (a–d) indicate no statistically significant differences (*p* < 0.05) based on ANOVA analysis and Tukey post-hoc test. Table S4. The effect of Ag NPs (at 20 mg/L) and AgNO3 solutions (at concentrations corresponding to 20 mg/L and 50 mg/L of Ag+ ions) on the content of polar metabolites in cotyledons of 4-day-old seedlings of pea (*Pisum sativum* L., cv. Tarchalska). Means of 3 replicates. The same letters by the values (a–d) indicate no statistically significant differences (*p* < 0.05) based on ANOVA analysis and Tukey post-hoc test. Table S5. The effect of seeds’ imbibition in AgNO3 solutions (at different concentrations—0 mg/L, 100 mg/L and 250 mg/L, for 8 h) on the length of root and epicotyl of 4-day-old seedlings of ten pea cultivars (including one fodder—‘Hubal’ and nine for general purpose). Values are means (*n* = 3). The same letters by the values (a-c for root, *a–c* for epicotyl) indicate no statistically significant differences (*p* < 0.05) based on ANOVA analysis and Tukey post-hoc test. Table S6. The concentration of total identified polar metabolites (TIPMs), including total soluble carbohydrates (TSCs), total amino acids (TPAAs), total organic acids (TOAs), and total remaining compounds (TRCs) in sprouts of 4-day-old seedlings of pea (normal, non-deformed seedlings—ND, deformed seedlings—D, *Pisum sativum* L., cv. Nemo) after pre-sowing treatment with AgNO3 at different concentrations. Means of 3 replicates. The same letters by the values (a–d) indicate no statistically significant differences (*p* < 0.05) based on ANOVA analysis and Tukey post-hoc test. Table S7. The concentration of total identified polar metabolites (TIPMs), including total soluble carbohydrates (TSCs), total amino acids (TPAAs), total organic acids (TOAs), and total remaining compounds (TRCs) in cotyledons of 4-day-old seedlings of pea (normal, non-deformed seedlings—ND, deformed seedlings—D, *Pisum sativum* L., cv. Nemo) after pre-sowing treatment with AgNO3 at different concentrations. Means of 3 replicates. The same letters by the values (a–d) indicate no statistically significant differences (*p* < 0.05) based on ANOVA analysis and Tukey post-hoc test.
