# Supplementary material for: The Imbibition of Pea (Pisum sativum L.) Seeds in Silver Nitrate Reduces Seed Germination, Seedlings Development and Their Metabolic Profile
Source: Plants (Basel). 2022 Jul 19;11(14):1877. doi: 10.3390/plants11141877 (PMC9323745; doi:10.3390/plants11141877)
Supplement: Supplementary file 1 [file plants-11-01877-s001.zip › plants-1766238-supplementary.pdf]

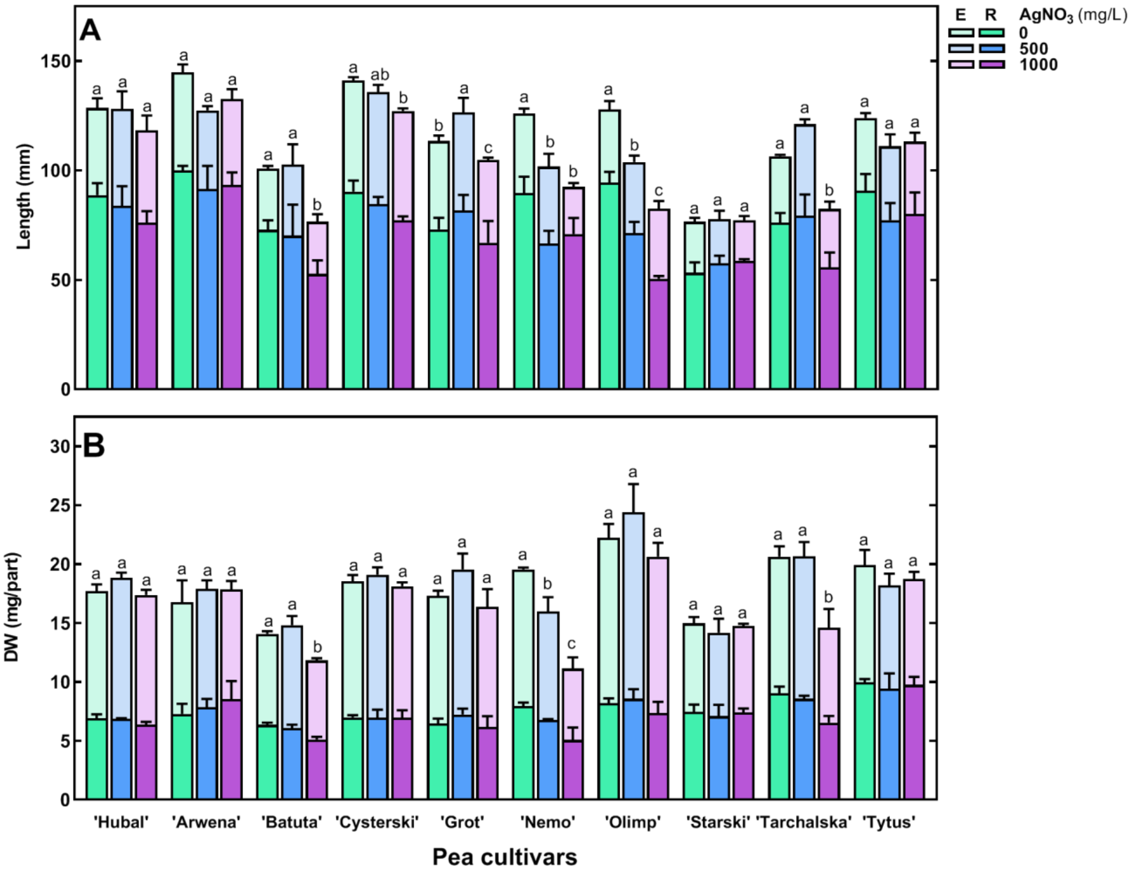

Figure S1. The effect of seeds imbibition in $AgNO_3$ solutions (at concentrations of 500 and 1000 mg/L, for 8 hours) on the length (**A**) and dry weight (**B**) of root (*R*) and epicotyl (*E*) of 4-day-old seedling of ten pea cultivars (including one fodder – 'Hubal' and nine for general-purpose). Values are means (n=3) + SD. Bars with the same letters (a-c) are not significantly (P<0.05) different (for a whole seedling) after ANOVA test and Tukey post hoc test.

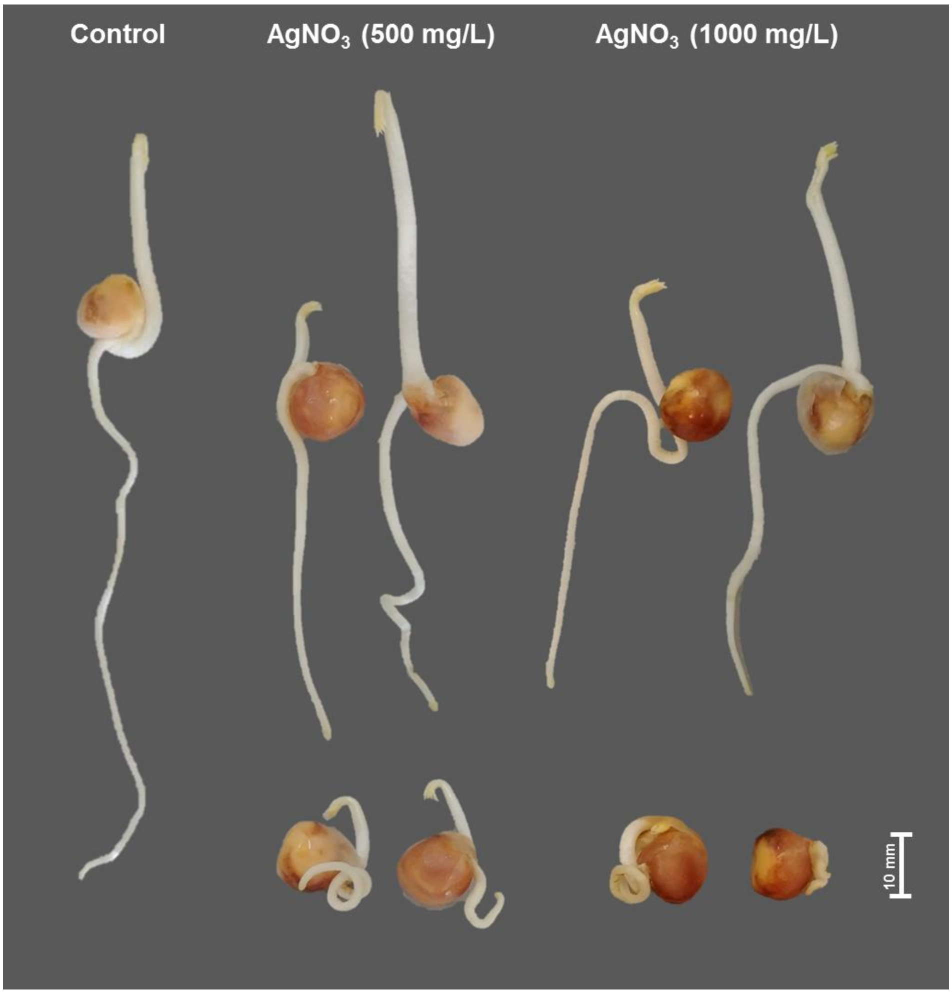

Figure S2. The effect of short-term imbibition of pea (*Pisum sativum* L. cv. Nemo) seeds in $AgNO_3$ solutions (at 500 and 1000 mg/L for 8 hours) on the morphology of 4-day-old seedlings. Control seedlings developed from seeds imbibed in double-distilled water. Normal, non-deformed seedlings - upper panel, deformed – lower.

Table S1. The parameters of polar metabolites identification in GC-MS analyses. RT – retention time, RI – retention index, ID – a type of identification of each metabolite – using mass spectra of original standards (S) or mass spectra from NIST 05 library (L), P – a percentage of similarity of identified metabolite to the standard metabolite and/or metabolite from the NIST library.

Metabolites	RT	RI	ID	P
Soluble carbohydrates				
fructose	13.527, 13.674	1917, 1935	S, L	94
galactose	13.774	1940	S, L	95
glucose	13.923, 14.151	1946, 1963	S, L	96
myo-inositol	16.806	2142	S, L	95
sucrose	23.082	2719	S, L	98
erythronic acid	9.271	1367	L	96
gluconic acid	15.357, 15.425	2023, 2028	L	95
raffinose	29.672	3466	S, L	98
stachyose	41.101	5053	S, L	98
Amino acids				
alanine	3.917	908	S, L	98
arginine	9.891	1427	L	92
asparagine	9.607, 10.680	1414, 1494	S, L	97
aspartic acid	8.665	1235, 1339	L	96
β-alanine	7.421	1248	L	94
GABA	8.772	1348	L	94
glutamic acid	9.958	1439	S, L	98
glutamine	11.988	1696	S, L	89
homoserine	7.770	1248	S, L	95
hydroxyproline	8.704	1342	L	96
isoleucine	5.809	1106	S, L	89
leucine	4.372	962	S, L	94
lysine	13.972	1929	S, L	95
methionine	8.615	1226	L	94
phenylalnine	10.071	1365, 1449	S, L	96
proline	4.573, 5.872	987, 1112	S, L	93
serine	5.385, 6.597	1066, 1173	S, L	96
threonine	6.940	1203	L	93
tyrosine	14.173	1313	S, L	95
valine	3.799, 4.971	897, 1025	S, L	96
Organic acids				
acetic acid	3.535	852	S, L	89
butyric acid	7.234	1225	L	87
citric acid	12.709	1847	S, L	90
fumaric acid	6.323	1152	S, L	89
glutaric acid	9.356	1368	L	89
lactic acid	3.579	871	S, L	91
malic acid	8.245	1306	S, L	96
malonic acid	4.818	989	L	92
oxalic acid	4.248	947	S, L	85
propionic acid	6.236	1142	L	91
succinic acid	5.976	1102	S, L	91
Other				
phosphoric acid	5.601	1087	S, L	92
urea	5.225	1044	L	92

Table S2. The effect of Ag NPs (at 20 mg/L) and $AgNO_3$ solutions (at concentrations corresponding to 20 and 50 mg/L of Ag^+ ions) on the content of polar metabolites in roots of 4-day-old seedlings of pea (*Pisum sativum* L., cv. Tarchalska). Means of 3 replicates. The same letters by the values (a-d) indicate no statistically significant differences ($P<0.05$) based on ANOVA analysis and Tukey post-hoc test.

	Water	Ag NPs (10nm)	Ag^+ ions	
		20 µg/mL	20 µg/mL	50 µg/mL
Metabolites	mg/g DW			
TIPMs, including:	101.31^{b}	136.45^{a}	99.09^{b}	95.21^{b}
TSCs, including:	33.51^{b}	50.97^{a}	27.55^{c}	32.98^{b}
fructose	0.84^{b}	2.46^{a}	0.99^{b}	1.16^{b}
galactose	1.20^{b}	3.98^{a}	1.74^{b}	1.49^{b}
glucose	3.57^{b}	17.28^{a}	0.92^{b}	3.35^{b}
myo-inositol	1.89^{b}	2.20^{a}	1.81^{b}	1.73^{b}
sucrose	25.59^{a}	24.27^{ab}	21.57^{b}	24.77^{a}
erythronic acid	0.27^{a}	0.47^{a}	0.30^{a}	0.25^{a}
gluconic acid	0.16^{c}	0.30^{a}	0.23^{b}	0.22^{b}
TAAs, including:	49.31^{bc}	59.22^{a}	53.59^{ab}	43.95^{c}
alanine	2.64^{a}	0.85^{c}	1.22^{b}	1.39^{b}
arginine	1.03^{a}	0.18^{b}	0.24^{b}	0.35^{b}
asparagine	3.91^{a}	2.78^{c}	2.78^{c}	3.28^{b}
aspartic acid	0.78^{a}	0.58^{a}	0.61^{a}	0.62^{a}
β-alanine	0.45^{a}	0.28^{c}	0.22^{d}	0.33^{b}
GABA	0.97^{b}	1.17^{a}	0.95^{b}	1.09^{a}
glutamic acid	1.51^{a}	1.49^{a}	1.03^{b}	1.37^{a}
glutamine	0.87^{a}	0.44^{b}	0.31^{b}	0.50^{b}
homoserine	28.11^{c}	45.12^{a}	34.66^{b}	28.49^{c}
hydroxyproline	0.79^{b}	1.10^{a}	0.84^{ab}	0.85^{ab}
isoleucine	0.69^{a}	0.52^{b}	0.59^{ab}	0.66^{a}
leucine	0.05^{a}	0.05^{a}	0.07^{a}	0.06^{a}
lysine	2.69^{b}	0.98^{c}	5.46^{a}	0.79^{c}
phenylalanine	0.47^{a}	0.30^{b}	0.45^{a}	0.45^{a}
proline	0.56^{a}	0.26^{c}	0.39^{b}	0.37^{b}
serine	1.86^{a}	1.36^{bc}	1.26^{c}	1.53^{b}
threonine	0.48^{ab}	0.57^{a}	0.47^{ab}	0.37^{b}
tyrosine	0.15^{b}	0.00^{b}	0.94^{a}	0.11^{b}
valine	1.30^{ab}	1.19^{bc}	1.11^{c}	1.33^{a}
TOAs, including:	10.20^{b}	16.72^{a}	9.90^{b}	10.21^{b}
acetic acid	0.13^{b}	0.50^{a}	0.15^{b}	0.15^{b}
butyric acid	0.09^{b}	0.20^{a}	0.19^{a}	0.08^{b}
citric acid	3.17^{b}	4.14^{a}	2.86^{b}	3.09^{b}
fumaric acid	0.10^{a}	0.14^{a}	0.10^{a}	0.10^{a}
glutaric acid	0.04^{a}	0.04^{a}	0.02^{a}	0.02^{a}
lactic acid	0.13^{b}	0.36^{a}	0.14^{b}	0.13^{b}
malic acid	5.84^{b}	10.25^{a}	5.78^{b}	6.02^{b}
malonic acid	0.02^{b}	0.04^{a}	0.02^{ab}	0.02^{b}
oxalic acid	0.13^{b}	0.41^{a}	0.15^{b}	0.13^{b}
propionic acid	0.15^{b}	0.26^{a}	0.19^{b}	0.15^{b}
succinic acid	0.39^{a}	0.38^{a}	0.30^{b}	0.34^{ab}
TRCs, including:	8.29^{b}	9.54^{a}	8.05^{b}	8.07^{b}
phosphoric acid	8.15^{b}	9.42^{a}	7.89^{b}	7.91^{b}
urea	0.14^{a}	0.13^{a}	0.16^{a}	0.15^{a}

Table S3. The effect of Ag NPs (at 20 mg/L) and $AgNO_3$ solutions (at concentrations corresponding to 20 and 50 mg/L of Ag^+ ions) on the content of polar metabolites in epicotyls of 4-day-old seedlings of pea (*Pisum sativum* L., cv. Tarchalska). Means of 3 replicates. The same letters by the values (a-d) indicate no statistically significant differences (P<0.05) based on ANOVA analysis and Tukey post-hoc test.

	Water	Ag NPs (10nm)	Ag^+ ions	
		20 μg/mL	20 μg/mL	50 μg/mL
Metabolites	mg/g DW			
TIPMs, including:	112.85[a]	103.99[b]	102.40[b]	101.53[b]
TSCs, including:	47.09[a]	36.72[b]	30.90[c]	35.74[b]
fructose	2.52[c]	3.23[a]	2.63[c]	2.94[b]
galactose	1.26[c]	2.22[a]	2.04[a]	1.64[b]
glucose	2.66[c]	4.12[a]	3.07[b]	3.39[b]
myo-inositol	2.81[a]	2.11[b]	2.10[bc]	2.02[c]
sucrose	37.36[a]	24.48[b]	20.56[c]	25.28[b]
erythronic acid	0.24[c]	0.35[a]	0.30[b]	0.28[b]
gluconic acid	0.24[a]	0.20[b]	0.21[b]	0.18[b]
TAAs, including:	48.32[b]	47.37[b]	52.30[a]	47.69[b]
alanine	1.88[a]	0.79[c]	1.11[b]	0.84[c]
arginine	0.62[a]	0.15[b]	0.12[b]	0.14[b]
asparagine	4.17[d]	4.52[c]	5.50[a]	4.87[b]
aspartic acid	0.52[a]	0.30[b]	0.30[b]	0.20[b]
β-alanine	0.40[a]	0.27[c]	0.23[d]	0.30[b]
GABA	2.36[a]	2.14[b]	2.11[b]	2.36[a]
glutamic acid	1.08[b]	1.17[a]	1.06[b]	1.11[ab]
glutamine	1.68[a]	1.00[c]	1.02[c]	1.23[b]
homoserine	26.89[c]	29.61[b]	32.38[a]	28.96[b]
hydroxyproline	0.94[bc]	0.86[c]	1.10[ab]	1.16[a]
isoleucine	0.73[a]	0.57[c]	0.66[b]	0.61[c]
leucine	0.03[b]	0.04[b]	0.06[a]	0.04[b]
lysine	2.24[a]	1.14[b]	0.79[c]	0.87[c]
phenylalanine	0.56[c]	0.49[d]	0.81[a]	0.63[b]
proline	0.47[a]	0.36[b]	0.46[a]	0.37[b]
serine	1.62[a]	1.32[c]	1.44[b]	1.45[b]
threonine	0.75[c]	1.34[a]	1.39[a]	1.12[b]
tyrosine	0.17[a]	0.13[a]	0.31[a]	0.12[a]
valine	1.22[bc]	1.16[c]	1.45[a]	1.31[b]
TOAs, including:	9.73[b]	11.78[a]	10.17[b]	10.01[b]
acetic acid	0.16[a]	0.17[a]	0.15[a]	0.17[a]
butyric acid	0.04[c]	0.12[a]	0.11[a]	0.08[b]
citric acid	3.45[b]	3.87[a]	3.10[c]	3.94[a]
fumaric acid	0.18[b]	0.23[a]	0.25[a]	0.24[a]
glutaric acid	0.02[d]	0.03[c]	0.08[a]	0.04[b]
lactic acid	0.29[a]	0.26[a]	0.25[a]	0.25[a]
malic acid	4.72[c]	6.16[a]	5.26[b]	4.43[c]
malonic acid	0.03[a]	0.03[ab]	0.02[b]	0.02[b]
oxalic acid	0.12[b]	0.16[ab]	0.16[a]	0.14[ab]
propionic acid	0.17[c]	0.22[ab]	0.25[a]	0.19[bc]
succinic acid	0.55[a]	0.53[a]	0.54[a]	0.50[b]
TRCs, including:	7.70[c]	8.12[b]	9.03[a]	8.08[b]
phosphoric acid	7.59[c]	8.03[b]	8.92[a]	8.00[b]
urea	0.11[a]	0.09[a]	0.11[a]	0.08[a]

Table S4. The effect of Ag NPs (at 20 mg/L) and $AgNO_3$ solutions (at concentrations corresponding to 20 and 50 mg/L of Ag^+ ions) on the content of polar metabolites in cotyledons of 4-day-old seedlings of pea (*Pisum sativum* L., cv. Tarchalska). Means of 3 replicates. The same letters by the values (a-d) indicate no statistically significant differences ($P<0.05$) based on ANOVA analysis and Tukey post-hoc test.

	Water	Ag NPs (10nm)	Ag^+ ions	
		20 µg/mL	20 µg/mL	50 µg/mL
Metabolites	mg/g DW			
TIPMs, including:	71.20^{b}	66.75^{b}	80.32^{a}	72.13^{b}
TSCs, including:	54.19^{ab}	49.24^{b}	59.03^{a}	53.93^{ab}
fructose	0.06^{a}	0.04^{a}	0.06^{a}	0.08^{a}
galactose	0.12^{a}	0.11^{a}	0.11^{a}	0.10^{a}
glucose	0.11^{a}	0.11^{a}	0.10^{a}	0.20^{a}
myo-inositol	1.77^{b}	1.74^{b}	2.04^{a}	1.75^{b}
sucrose	47.02^{b}	42.50^{b}	52.31^{a}	46.43^{b}
raffinose	1.08^{b}	1.11^{b}	1.06^{b}	1.35^{a}
stachyose	4.03^{a}	3.62^{ab}	3.35^{b}	4.02^{a}
TAAs, including:	11.32^{b}	11.60^{b}	14.96^{ab}	12.87
alanine	0.57^{b}	0.62^{b}	0.77^{a}	0.63^{b}
arginine	1.58^{a}	0.96^{b}	1.08^{b}	1.02^{b}
asparagine	0.52^{ab}	0.45^{b}	0.57^{ab}	0.64^{a}
β-alanine	0.10^{c}	0.11^{b}	0.13^{a}	0.11^{b}
GABA	0.98^{b}	1.26^{a}	1.33^{a}	0.82^{c}
glutamic acid	0.79^{c}	0.95^{b}	1.46^{a}	1.41^{a}
glutamine	1.04^{a}	0.60^{b}	0.96^{a}	0.95^{a}
homoserine	2.09^{a}	2.62^{a}	2.71^{a}	2.15^{a}
hydroxyproline	0.58^{d}	0.72^{c}	1.03^{a}	0.93^{b}
isoleucine	0.15^{c}	0.20^{b}	0.29^{a}	0.26^{a}
lysine	1.10^{a}	0.77^{b}	1.05^{a}	1.02^{a}
phenylalanine	0.11^{c}	0.19^{bc}	0.38^{a}	0.25^{b}
proline	0.35^{d}	0.44^{c}	0.70^{a}	0.55^{b}
serine	0.68^{d}	0.94^{c}	1.34^{a}	1.09^{b}
threonine	0.08^{b}	0.10^{b}	0.15^{a}	0.14^{a}
tyrosine	0.29^{b}	0.20^{c}	0.31^{ab}	0.34^{a}
valine	0.32^{d}	0.46^{c}	0.71^{a}	0.56^{b}
TOAs, including:	4.15^{a}	4.27^{a}	4.18^{a}	3.56^{b}
citric acid	3.13^{ab}	3.22^{a}	3.00^{b}	2.60^{c}
fumaric acid	0.03^{a}	0.03^{a}	0.03^{a}	0.03^{a}
lactic acid	0.22^{bc}	0.24^{b}	0.31^{a}	0.19^{c}
malic acid	0.38^{a}	0.34^{a}	0.30^{a}	0.31^{a}
malonic acid	0.03^{b}	0.03^{b}	0.04^{a}	0.04^{a}
propionic acid	0.03^{b}	0.04^{ab}	0.04^{a}	0.03^{c}
succinic acid	0.34^{b}	0.37^{b}	0.47^{a}	0.37^{b}
TRCs, including:	1.53^{c}	1.64^{bc}	2.15^{a}	1.76^{b}
phosphoric acid	1.50^{c}	1.61^{bc}	2.11^{a}	1.73^{b}
urea	0.03^{b}	0.03^{b}	0.04^{a}	0.03^{b}

Table S5. The effect of seeds imbibition in $AgNO_3$ solutions (at different concentrations – 0, 100 and 250 mg/L, for 8 hours) on the length of root and epicotyl of 4-day-old seedling of ten pea cultivars (including one fodder – 'Hubal' and nine for general-purpose). Values are means (n=3). The same letters by the values (a-c for root, *a-c* for epicotyl) indicate no statistically significant differences (P<0.05) based on ANOVA analysis and Tukey post-hoc test.

Cultivar	$AgNO_3$ concentration					
	0 mg/L		100 mg/L		250 mg/L	
	Root	Epicotyl	Root	Epicotyl	Root	Epicotyl
	Length (mm)					
Hubal	88.67^{a}	39.85^{a}	85.24^{a}	40.77^{a}	84.82^{a}	38.23^{a}
Arwena	100.83^{a}	44.77^{a}	96.15^{a}	39.56^{a}	93.42^{a}	38.57^{a}
Batuta	72.83^{a}	27.97^{b}	71.81^{a}	28.21^{ab}	70.45^{a}	32.56^{a}
Cysterski	90.28^{a}	50.74^{a}	87.15^{a}	50.33^{a}	86.22^{a}	51.22^{a}
Grot	73.04^{b}	40.39^{b}	74.73^{b}	42.50^{a}	76.17^{a}	43.77^{a}
Nemo	89.64^{a}	36.49^{b}	90.33^{a}	43.31^{a}	59.16^{b}	33.94^{c}
Olimp	94.49^{a}	33.33^{a}	88.15^{b}	33.75^{a}	76.40^{c}	32.28^{a}
Starski	53.22^{a}	23.32^{a}	54.17^{a}	22.50^{a}	55.56^{a}	20.87^{a}
Tarchalska	76.13^{b}	30.39^{b}	88.83^{a}	40.05^{a}	74.84^{b}	38.97^{a}
Tytus	90.74^{a}	33.23^{a}	86.28^{a}	33.33^{a}	84.59^{a}	33.21^{a}

Table S6. The concentration of total identified polar metabolites (TIPMs), including total soluble carbohydrates (TSCs), total amino acids (TPAAs), total organic acids (TOAs), and total remaining compounds (TRCs) in sprouts of 4-day-old seedlings of pea (normal, non-deformed seedlings – ND, deformed seedlings – D, *Pisum sativum* L., cv. Nemo) after pre-sowing treatment with $AgNO_3$ at different concentrations. Means of 3 replicates. The same letters by the values (a-d) indicate no statistically significant differences (P<0.05) based on ANOVA analysis and Tukey post-hoc test.

	$AgNO_3$ concentration				
	0 mg/L	500 mg/L		1000 mg/L	
		ND	D	ND	D
Metabolites	mg/g DW				
TIPMs, including:	120.67^{a}	120.83^{a}	105.88^{b}	112.84^{ab}	118.49^{ab}
TSCs, including:	51.24^{b}	59.22^{a}	42.16^{c}	52.82^{b}	51.47^{b}
fructose	4.58^{a}	4.29^{a}	0.65^{c}	3.81^{a}	2.24^{b}
galactose	8.23^{a}	8.11^{a}	0.51^{c}	6.68^{a}	2.70^{b}
glucose	14.31^{b}	17.59^{a}	1.79^{d}	15.06^{b}	5.83^{c}
myo-inositol	2.50^{a}	2.44^{a}	1.94^{b}	2.32^{ab}	2.62^{a}
sucrose	21.15^{b}	26.18^{b}	37.02^{a}	24.29^{b}	37.73^{a}
erythronic acid	0.29^{b}	0.42^{a}	0.10^{c}	0.36^{ab}	0.15^{c}
gluconic acid	0.17^{b}	0.19^{b}	0.14^{b}	0.30^{a}	0.21^{b}
TAAs, including:	47.13^{a}	37.31^{b}	37.06^{b}	36.60^{b}	39.90^{b}
alanine	1.10^{b}	1.24^{b}	4.25^{a}	1.17^{b}	3.82^{a}
arginine	0.40^{c}	0.30^{c}	2.14^{a}	0.26^{c}	1.22^{b}
asparagine	6.01^{a}	4.15^{b}	3.56^{c}	4.01^{bc}	3.60^{bc}
aspartic acid	0.15^{b}	0.11^{b}	0.24^{a}	0.12^{b}	0.14^{b}
β-alanine	0.20^{d}	0.28^{c}	0.59^{a}	0.23^{cd}	0.48^{b}
GABA	1.23^{c}	1.83^{b}	3.02^{a}	1.82^{b}	2.75^{a}
glutamic acid	0.98^{b}	0.94^{b}	2.39^{a}	0.93^{b}	2.03^{a}
glutamine	0.83^{d}	1.04^{cd}	2.30^{a}	1.23^{c}	1.67^{b}
homoserine	28.11^{a}	20.13^{b}	8.66^{d}	19.92^{b}	14.83^{c}
hydroxyproline	0.85^{c}	0.89^{c}	2.30^{a}	0.93^{c}	1.85^{b}
isoleucine	0.66^{c}	0.68^{bc}	0.85^{a}	0.63^{c}	0.79^{ab}
leucine	0.05^{c}	0.09^{a}	0.06^{b}	0.09^{ab}	0.05^{c}
lysine	1.35^{a}	0.90^{b}	1.34^{a}	0.91^{b}	1.36^{a}
phenylalanine	0.65^{a}	0.42^{bc}	0.48^{bc}	0.35^{c}	0.51^{b}
proline	0.62^{c}	0.72^{c}	1.14^{a}	0.61^{c}	0.94^{b}
serine	1.48^{ab}	1.26^{c}	1.35^{bc}	1.17^{c}	1.56^{a}
threonine	1.04^{a}	0.98^{a}	0.45^{b}	0.92^{a}	0.55^{b}
tyrosine	0.12^{c}	0.09^{c}	0.80^{a}	0.16^{c}	0.57^{b}
valine	1.29^{a}	1.26^{a}	1.14^{a}	1.12^{a}	1.17^{a}
TOAs, including:	14.07^{a}	15.93^{a}	16.20^{a}	14.37^{a}	16.86^{a}
acetic acid	0.18^{a}	0.17^{a}	0.17^{a}	0.18^{a}	0.17^{a}
butyric acid	0.22^{a}	0.10^{c}	0.02^{d}	0.12^{b}	0.01^{d}
citric acid	5.52^{abc}	6.55^{a}	5.27^{bc}	5.83^{ab}	4.61^{c}
fumaric acid	0.20^{bc}	0.28^{a}	0.15^{d}	0.23^{ab}	0.18^{cd}
glutaric acid	0.03^{a}	0.03^{a}	0.02^{b}	0.03^{a}	0.02^{b}
lactic acid	0.18^{bc}	0.17^{c}	0.24^{ab}	0.18^{bc}	0.27^{a}
malic acid	6.72^{b}	7.48^{b}	9.19^{a}	6.69^{b}	10.35^{a}
malonic acid	0.05^{b}	0.05^{b}	0.08^{a}	0.04^{b}	0.08^{a}
oxalic acid	0.17^{a}	0.17^{a}	0.14^{a}	0.18^{a}	0.15^{a}
propionic acid	0.27^{a}	0.30^{a}	0.12^{c}	0.27^{a}	0.17^{b}
succinic acid	0.52^{c}	0.65^{bc}	0.79^{ab}	0.60^{c}	0.85^{a}
TRCs, including:	8.24^{b}	8.36^{b}	10.46^{a}	9.05^{ab}	10.26^{a}
phosphoric acid	8.14^{b}	8.29^{b}	10.21^{a}	8.97^{ab}	10.06^{a}
urea	0.10^{b}	0.07^{b}	0.26^{a}	0.07^{b}	0.20^{a}

Table S7. The concentration of total identified polar metabolites (TIPMs), including total soluble carbohydrates (TSCs), total amino acids (TPAAs), total organic acids (TOAs), and total remaining compounds (TRCs) in cotyledons of 4-day-old seedlings of pea (normal, non-deformed seedlings – ND, deformed seedlings – D, *Pisum sativum* L., cv. Nemo) after pre-sowing treatment with $AgNO_3$ at different concentrations. Means of 3 replicates. The same letters by the values (a-d) indicate no statistically significant differences (P<0.05) based on ANOVA analysis and Tukey post-hoc test.

	$AgNO_3$ concentration				
	0 mg/L	500 mg/L		1000 mg/L	
		ND	D	ND	D
Metabolites	mg/g DW				
TIPMs, including:	67.65[a]	63.27[ab]	57.81[b]	66.32[a]	46.74[c]
TSCs, including:	48.61[a]	47.50[a]	47.59[a]	49.62[a]	38.95[b]
fructose	0.04[d]	0.05[cd]	0.09[ab]	0.07[bc]	0.09[a]
galactose	0.07[b]	0.08[b]	0.06[b]	0.04[b]	0.20[a]
glucose	0.11[c]	0.12[bc]	0.13[bc]	0.18[a]	0.14[b]
myo-inositol	1.98[ab]	2.00[ab]	1.85[bc]	2.13[a]	1.67[c]
sucrose	41.77[a]	40.14[ab]	36.22[b]	41.62[a]	29.33[c]
raffinose	1.06[c]	1.17[c]	2.59[a]	1.31[c]	2.05[b]
stachyose	3.57[c]	3.94[c]	6.65[a]	4.28[c]	5.47[b]
TAAs, including:	14.16[a]	12.11[b]	6.28[c]	12.57[ab]	4.29[d]
alanine	0.70[a]	0.47[b]	0.75[a]	0.39[b]	0.49[b]
arginine	0.86[a]	0.82[a]	0.41[b]	0.76[a]	0.17[c]
asparagine	0.61[a]	0.47[b]	0.30[c]	0.51[b]	0.19[d]
β-alanine	0.17[a]	0.05[c]	0.04[c]	0.09[b]	0.04[c]
GABA	2.12[a]	0.69[c]	0.58[cd]	0.95[b]	0.44[d]
glutamic acid	0.61[c]	1.12[a]	1.05[a]	0.82[b]	0.61[c]
glutamine	1.25[b]	1.70[a]	0.57[c]	1.60[a]	0.42[c]
homoserine	2.63[a]	1.50[b]	0.16[c]	1.82[b]	0.08[c]
hydroxyproline	0.65[b]	0.90[a]	0.39[c]	0.83[a]	0.29[d]
isoleucine	0.21[b]	0.25[ab]	0.11[c]	0.28[a]	0.11[c]
lysine	1.01[a]	1.11[a]	0.49[b]	1.16[a]	0.31[c]
methionine	0.04[a]	0.07[a]	0.04[a]	0.06[a]	0.04[a]
phenylalanine	0.34[a]	0.18[c]	0.04[d]	0.27[b]	0.02[d]
proline	0.76[a]	0.76[a]	0.39[b]	0.75[a]	0.49[b]
serine	1.14[a]	1.01[b]	0.37[c]	1.10[ab]	0.21[d]
threonine	0.11[a]	0.09[b]	0.07[c]	0.11[a]	0.04[d]
tyrosine	0.34[b]	0.45[a]	0.28[b]	0.52[a]	0.19[c]
valine	0.61[a]	0.48[b]	0.21[c]	0.56[a]	0.15[d]
TOAs, including:	2.74[a]	2.20[c]	2.58[ab]	2.33[bc]	2.39[bc]
citric acid	1.29[b]	1.58[a]	1.62[a]	1.58[a]	1.42[ab]
fumaric acid	0.03[a]	0.02[a]	0.03[a]	0.03[a]	0.03[a]
lactic acid	0.46[a]	0.01[c]	0.18[b]	0.04[c]	0.18[b]
malic acid	0.24[b]	0.19[b]	0.44[a]	0.20[b]	0.48[a]
malonic acid	0.06[b]	0.07[a]	0.05[b]	0.07[a]	0.05[b]
propionic acid	0.05[a]	0.03[b]	0.01[c]	0.03[b]	0.01[c]
succinic acid	0.61[a]	0.29[c]	0.26[c]	0.38[b]	0.23[c]
TRCs, including:	2.15[a]	1.46[c]	1.36[c]	1.80[b]	1.10[d]
phosphoric acid	2.12[a]	1.46[c]	1.34[c]	1.79[b]	1.08[d]
urea	0.03[a]	0.01[c]	0.03[ab]	0.01[c]	0.02[b]
